# On Cystic Oxide, a New Species of Urinary Calculus

**Published:** 1811-03

**Authors:** William Hyde Wollaston


### *On Cystic Oxide, a new Species of Urinary Calculus.* By William Hyde Wollaston, M. D. Sec. R. S.

[Phil. Trans. Part 2. 1810.]

THE principal design of the present essay is to make known the existence, and to describe the leading properties, of a new species of urinary calculus from the human bladder; but I shall at the same time take the opportunity of correcting an inaccuracy or two that I have observed in my former communication on this subject. (Phil. Trans. 1797.)

I on that occasion took notice of five kinds of urinary calculi.

1. The *lithic acid*, since called *uric acid*, originally analysed by Scheele.

2. The

2. The *oxalate of lime*, or *mulberry calculus*.

3. The phosphate of lime, or *bone-earth calculus*.

4. The ammoniacal phosphate of magnesia.

5. The *fusible calculus*, which consists of the two last species combined.

It is now about five years since I first met with another species, evidently differing from each of those before described. It was in the possession of Dr. Reeve, of Norwich, who obligingly gave me a portion of it for the purpose of examining its chemical qualities. It had been taken from his brother when he was five years old, and at that time was covered with a coating of phosphate of lime very loose in its texture, and consequently very soon separated.* This species is probably very rare; for, although I have omitted no opportunity of paying attention to any urinary concretions to which I could have access, I have, to this time, seen only one other specimen of the same substance. This last is in a collection of calculi belonging to Guy's Hospital, given by Mr. Lucas, surgeon to that institution, having been formed partly by his father, and partly by himself, in the course of their practice; and according to the present arrangement, (which, it is hoped, will not be altered) the calculus to which I allude may be found by reference to No. 46 of that collection. It was extracted by the usual operation, from a man of 36 years of age, of whom no record is preserved, except that his name was William Small. It weighed, when entire, 270 grains.

In appearance, these calculi resemble more nearly the triple phosphate of magnesia, than any other calculus; but they are more compact than that compound is usually found to be: not consisting of distinct laminæ, but appearing as one mass confusedly crystallized throughout its substance. Hence, instead of having the opacity and whiteness observable in fusible calculi, which consist of a number of small crystals cemented together, these calculi have a yellowish semi-transparency; and they have also a peculiar glistening lustre, like that of a body having a high refractive density.

When this substance is submitted to destructive distilla-

* I am informed that another stone formed afterwards in the bladder of this boy, and that he died in consequence, without submitting to the operation a second time. The stone found in his bladder after death, consisted principally of uric acid, but was peculiar in one respect, as its centre was hollow by the removal of some more soluble substance, of which the nucleus had consisted.

tion tion, it yields fœtid carbonate of ammonia, partly fluid, and partly in a solid state, and a heavy fœtid oil, such as usually proceeds from animal substances; and there remains a black spongy coal, much smaller in proportion than is found after the distillation of uric calculi.

Under the blow-pipe it may be distinguished from uric acid by the smell, which at no period resembles that of prussic acid; but in addition to the usual smell of burnt animal substances, there is a peculiar fœtor, of which I cannot give a correct idea, as I know no smell which it can be said to resemble.

This species of calculus is so readily acted upon by the generality of common chemical agents, that its character may perhaps be most distinctly marked, by an enumeration of those feeble powers that it can resist.

It is not dissolved (excepting in very small proportion) by water, by alcohol, by acetic acid, by tartaric acid, by citric acid, or by saturated carbonate of ammonia.

The solvents, on the contrary, are far more numerous. It is dissolved, in considerable quantity, by muriatic acid, by nitric acid, by sulphuric acid, by phosphoric acid, and by oxalic acid.

It is also dissolved readily by pure alkaline menstrua: by potash, by soda, by ammonia, and by lime-water. It is even dissolved by fully saturated carbonates of potash or of soda. Accordingly, these alkalies are not so convenient for the precipitation of this matter from acid solutions, as the carbonate of ammonia, which is not capable of redissolving the precipitate, though added in excess.

For a similar reason, the acid best suited for its precipitation from alkaline solutions, are the acetic and citric acids. But the tartaric acid may occasion an appearance of precipitation, by forming a supertartrate with the alkali employed.

The combination of this substance with acids may be made to crystallize without difficulty, and they form slender spicula radiating from a centre, which readily dissolve again in water, unless they have been injured by being in any degree over-heated.

The muriatic salt is decomposed by the heat of boiling water, on account of the volatility of the acid, and the rest are easily destroyed by a greater excess of heat.

The salt formed by combination with nitric acid, does not yield oxalic acid, and does not become red, as the uric acid does, when similarly treated; but it turns brown, becoming gradually darker, till it is ultimately black.

When the combinations with alkalies are evaporated, they leave leave small granular crystals; but as I was desirous of rendering my experiments as numerous as a limited quantity would permit, the portion which I could employ in any one experiment was too small for me to attempt to determine the form of such crystals.

When a hot solution in potash was neutralized by distilled vinegar, the precipitate did not immediately take place, but formed gradually during the cooling of the liquor in minute crystals, some at the surface of the fluid, and others attached to the sides of the vessel. The only definite form which I could observe, was that of flat hexagonal plates, but I could discern nothing which enabled me to judge of the primitive form of the crystal. On the surface of the calculus belonging to Guy's hospital, some minute crystals may be discerned, of a different shape, being nearly cubic. And it is possible, that the hexagonal crystals may owe their figure to a small portion of alkali remaining in combination.

From the ready disposition of this substance to unite with both acids and alkalies, it would appear to be an oxide; and that it does, in fact, contain oxygen, is proved by the formation of carbonic acid in distillation. The quantity of oxygen present in the calculus is not, however, sufficient to give it acid properties, for it has no effect on paper coloured with litmus.

I am therefore inclined to consider it as an oxide: and since both the calculi that have yet been observed have been taken from the bladder, it may be convenient to give it the name of *cystic oxide*, which will serve to distinguish it from other calculi; and as this is unlike any other term at present employed in chemistry, it is to be hoped that it will not be thought to require any alteration.

Since the period of my first essay on gouty and urinary concretions, the general results contained in it have been confirmed by others, and I believe are incontrovertible. But I am under the necessity of acknowledging a mistake in the analysis of the mulberry calculus, though not of much importance. An acid is mentioned to have arisen by sublimation, and it was supposed to originate from a partial decomposition of the oxalic acid. But since pure oxalate of lime yields no such sublimate, it most probably arose from the mixture of a small quantity of uric acid in the calculus then under examination.

In the analysis of the triple phosphate of magnesia, there is another mistake of more consequence. In my selection from numerous experiments for ascertaining the presence of phosphoric acid, I gave the preference to one in which nitrate of mercury was employed, on account of the facility of extracting the acid from the phosphate of mercury, by heat alone. But since the whole of the phosphoric acid is not precipitated by nitrate of mercury, sulphate of magnesia will not be formed on the addition of sulphuric acid, and the magnesia cannot be obtained separate by the same process.

It may have been in consequence of this oversight, that a mistake on that subject has occurred in the succeeding volume of the Transactions.

A calculus is there described, which had been taken by Mr. Thomas from the bladder of a dog, and a series of experiments are related, from which it was inferred to consist of super-phosphate of lime, and phosphate of ammonia. But from the appearance of this calculus (which was exhibited to the Society at the time when the paper was read) I was much inclined to think that the nature of it was mistaken, and upon full consideration of the experiments, they did not appear to me conclusive.

I therefore obtained a portion of the calculus, and by the following process, the earth contained in it was proved to consist almost wholly of magnesia.

It was dissolved, with the exception of a very small residuum, by distilled vinegar.

The whole of the phosphoric acid was then precipitated by acetate of lead, added to excess.

The liquor was then poured off, and sulphuric acid was added, which precipitated the excess of lead, and at the same time formed sulphate of magnesia in solution.

By evaporation to dryness, the acetic acid was removed, and by subsequent increase of heat, the sulphate of ammonia and excess of sulphuric acid were expelled.

The residuum being then dissolved in water, and the liquor suffered to crystallize by spontaneous evaporation, there remained a quantity of sulphate of magnesia, that weighed rather more than the quantity of calculus taken for the experiment.

It was evident, therefore, that in this instance, the calculus examined did not consist of super-phosphate of lime, and there is some reason to doubt, whether a compound, that is so very soluble in water, ever forms a part of urinary concretions.

Although the treatment of diseases is not in general a fit subject to occupy the time of this Society, there is nevertheless one suggestion, with respect to the prevention of calculous complaints, so nearly connected with my present subject, that I think it may deserve to be recorded.

Since the white matter contained in the urine of birds, which which is voided along with their dung, has been remarked by M. Vauquelin to consist principally of uric acid, I have paid some attention to the different proportion in which this matter is voided by different species of birds, to see how far it accorded with the different qualities of their food. And I found that in the dung of the goose, feeding wholly on grass, the proportion did not seem so much as $\frac{1}{200}$ of the whole dung. In that of a pheasant kept in a cage, and fed on barley alone, it was about $\frac{1}{14}$ part. In that of a hen, having the range of a garden and farm-yard, and consequently procuring insects, and possibly other animal food, the proportion was manifestly much greater, and combined with lime. In the dung of a hawk, fed upon flesh alone, the quantity of matter voided in a solid state bears but a small proportion to the residuum of uric acid that is left by the urine when dry. And in the gannet feeding solely on fish, I have observed the evacuations in some instances to be mere urine, for it contained no solid matter, excepting the uric acid.

It seems consequently deserving of inquiry, what changes might be produced in the urine of any one animal, by such alterations of diet, as its constitution would permit; for as far as any inference can be drawn from these varieties, which naturally occur, it would appear, that persons subject to calculi consisting of uric acid, as well as gouty persons, in whom there is always a redundance of the same matter, have much reason to prefer vegetable diet; but that the preference usually given to fish above other kinds of animal food, is probably erroneous.

